# Evaluating the Effect of Expressing a Peanut Resveratrol Synthase Gene in Rice

**DOI:** 10.1371/journal.pone.0136013

**Published:** 2015-08-24

**Authors:** Shigang Zheng, Shanchang Zhao, Zhen Li, Qingguo Wang, Fangyin Yao, Lianqun Yang, Jiaowen Pan, Wei Liu

**Affiliations:** 1 Department of Bio-Tech Research Center, Shandong Academy of Agricultural Sciences, Department of Key Laboratory of Genetic Improvement, Ecology and Physiology of Crops, Shandong Province, Jinan, Shandong, People's Republic of China; 2 Department of Agricultural Quality Standards and Testing Technology, Shandong Academy of Agricultural Sciences, Jinan, Shandong, People's Republic of China; 3 Department of Life Science, Qingdao Agricultural University, Tsingtao, Shandong, People's Republic of China; Northeast Forestry University, CHINA

## Abstract

Resveratrol (Res) is a type of natural plant stilbenes and phytoalexins that only exists in a few plant species. Studies have shown that the Res could be biosynthesized and accumulated within plants, once the complete metabolic pathway and related enzymes, such as the key enzyme resveratrol synthase (RS), existed. In this study, a RS gene named *PNRS1* was cloned from the peanut, and the activity was confirmed in *E*. *coli*. Using transgenic approach, the *PNRS1* transgenic rice was obtained. In T_3_ generation, the Res production and accumulation were further detected by HPLC. Our data revealed that compared to the wild type rice which *trans*-resveratrol was undetectable, in transgenic rice, the *trans*-resveratrol could be synthesized and achieved up to 0.697 μg/g FW in seedlings and 3.053 μg/g DW in seeds. Furthermore, the concentration of *trans*-resveratrol in transgenic rice seedlings could be induced up to eight or four-fold higher by ultraviolet (UV-C) or dark, respectively. Simultaneously, the endogenous increased of Res also showed the advantages in protecting the host plant from UV-C caused damage or dark-induced senescence. Our data indicated that Res was involved in host-defense responses against environmental stresses in transgenic rice. Here the results describes the processes of a peanut resveratrol synthase gene transformed into rice, and the detection of *trans*-resveratrol in transgenic rice, and the role of *trans*-resveratrol as a phytoalexin in transgenic rice when treated by UV-C and dark. These findings present new outcomes of transgenic approaches for functional genes and their corresponding physiological functions, and shed some light on broadening available resources of Res, nutritional improvement of crops, and new variety cultivation by genetic engineering.

## Introduction

Resveratrol (3,4′,5-trihydroxy-stilbene, Res) is a non-flavonoid polyphenolic compound in plant phenylpropanoide related secondary metabolism. It has been characterized as a very important phytoalexin [1] and antiradical component [2] in plants, which has protecting function in both animals and plants, such as antipathogen [3], antioxidant activities [4], cancer prevention [5], inflammation inhibition [6], and life span extension [7]. However, it has only been reported to exist in a few plant species, including grape [8], peanut [9], *Polygonum cuspidatum* [10], *Parthenocissus henryane* [11], and sorghum [12].

It is known that the Res biosynthesis pathway belongs to the phenylalanine metabolic pathways, which four key enzymes of phenylalanine ammonialyase (PAL), cinnamate-4-hydroxylase (C4H), 4-coumarate-CoA ligase (4CL), and resveratrol synthase (RS) were involved, and the genes of these four enzymes had been identified and cloned. As there has a branch of synthetic way, and two of type III polyketide synthase such as RS and chalcone synthase (CHS) have competitive effects, the eventually metabolites of the pathway mainly depends on the competition of RS and CHS, and only the synthetic way catalyzed by RS could synthesis Res [13]. In many plant species such as rice, it is konwn to contain the synthetic substrates of Res, such as coumaryl Co-A and malonyl Co-A, but lack of the key synthetic enzyme of RS, so it is still unable to synthesize Res within the plants [14]. Since there are such an important role of the RS gene in the Res synthesis process, so it has been intensively and globally investigated [15, 16]. Resports showed that the RS gene from *Arachis hypogaea* that transformed into *Nicotiana tabacum* cold lead to the production of Res [17], and many other RS genes of different species have also been transformed into various plant species and microorganisms [14], which suggested that genetic engineering in this field is feasible and effective. Heterogeneous transformation of RS genes from *Vitis vinifera* has been frequently reported, which are known as *vst1* [18], *StSy* [19], and *PSV25* [20]. The RS genes cloned from peanut have been successively transformed into alfalfa [21], *Rehmannia glutinosa* [22], and purple sweet potato [23]. In view of the existence of endogenous processing and modification, the metabolites of *trans*-resveratrol and its isomers or derivates could be newly synthesized and detected in transgenic plants.

Rice (*Oryza sativa* L.), one of the important crops, is reported to contain the enzymes required for Res biosynthesis except for RS, so that it can only synthesize and produce flavonoids instead of Res [24]. The RS gene *vst1* of grape was initially used to transform the rice *japonica* cultivar Nipponbare [25], and then widely applied and transformed into *japonica* rice cultivars such as Zhonghua 8, 9, 10, 13, Zhongbai 4, and indica-type 8706 [26]. Although the heterogeneous transformations were confirmed and the exogenous genes were integrated into rice genome, the synthesis and accumulation of new secondary metabolites, such as Res or its derivates, are still not detected [16]. Recently, Baek et al. reported the production of Res in the transgenic rice cultivar Dongjin, transformed with *AhSTS1* [27]. Meantime, for more insights into the functions of RS gene and Res, we isolated the *PNRS1* from peanut cultivar Luhua14 and transformed it into the rice cultivar Shengdao13 by *Agrobacterium*-mediated transformation [28].

Early researches mainly focused on the antipathogen abilities in Res accumulated plants, such as against *Botrytis cinerea* [3, 18], *Pyricularia oryzae* [25], *Phoma medicaginis* [21], and *Colletotrichum higginsianum* [10]. Some studies also showed that the abiotic stress resistance was improved in Res accumulated plants [8, 9]. Although there has remain query of the specific mechanisms of antipathogen abilities of Res, a recent report showed that Res resistance to ROS is one aspect involved in the stress resistant processes [1]. ROS damages in plants caused by growth and environmental factors, such as UV radiation and senescence, can be reduced by endogenous or exogenous Res [9, 29]. At present, reports that show the improvement of tolerance to abiotic stress in Res contained plants and the benefits of Res enriched diets on animals or humans, are receiving more attention [27, 30]. The production of Res through genetic engineering in crops, such as tomato and rice, has been reported to be helpful in protecting the heart, inhibiting cyclo-oxigenase-2 enzyme activity, and preventing metabolic syndrome in mice [6, 27].

In this paper, we confirmed the RS enzyme activity of *PNRS1* encoded protein in *Escherichia Coli*. Also, the rice T_3_ generations of homozygous lines were harvested, and eight independent lines were further selected for secondary metabolic analysis by HPLC. The result shows that *trans*-resveratrol is produced and accumulated in the seedlings and seeds of transgenic rice, and is induced by UV-C and dark treatments. The transgenic plants display less damage symptoms compared with those of wild-type rice, which indicates the involvement of *PNRS1* in stress resistance responses of transgenic rice.

## Materials and Methods

### Plant materials and growth conditions

The rice cultivar Shengdao13 (*Oryza sativa* L.) was used in this paper. For laboratory investigation, the rice seeds were first soaked for two days for germination, and then were cultured in basal medium and transferred into a climatic chamber (28°C, 75% RH, 16-h light/8-h dark cycles) for a further two weeks growth. The germinated rice seeds were transplanted into soil and grown in a greenhouse for preparation of six-week-old rice leaves.

### *PNRS1* Cloning and binary vector construction

The cloning of *PNRS1* (GenBank: FM955393), construction of binary vector of pCA1300-Ubi-PNRS1 and genetic transformation, were performed as previously described [28]. Gene specific primers for *PNRS1* were PNRS-F: 5′-CGGGATCCATGGTGTCTGTGA GTGGAATT-3′ and PNRS-R: 5′-CGAGCTCGTATTATATGGCCATGCTGC-3′. The binary vector of pCA1300-Ubi-PNRS1 was constructed using pCAMBIA1300 as the framework with insertion of an expression cassette encoding the maize *Ubi* promoter [31] and herbicide resistance *bialaphos (bar)* gene as selective marker instead of the original 35S promoter and hygromycin gene.

### Identification of transgenic rice

As *bar* gene was used as the selective marker, the screening of the transgenic plants was carried out by spraying the seedlings with 1 g/l basta. Further identification of positive transformants was implemented by molecular detection using genomic DNA of transformants extracted by the TPS method [32] as templates. The PCR products were analyzed by agarose gel electrophoresis, using plasmid pCA1300-Ubi-PNRS1 as a positive control. At T_0_ generation, six transgenic plants containing the *PNRS1* fragment were selected from seven transgenics resistant to basta. After screening of the T_1_ and T_2_ generation, the T_3_ generation was harvested and used for detailed physiological and biochemical analysis.

### Semi-quantitative PCR analysis

Total RNA was isolated from the leaves of T_3_ transgenic rice using TransZol reagent (TransGen Biotech; China). First-strand cDNA was synthesized from 2 μg of total RNA using the PrimeScript П 1st strand cDNA synthesis kit (Takara Biotech; Japan), and the generated cDNA served as a template for reverse transcription polymerase chain reaction (RT-PCR) amplification using *actin* of rice as the internal control. The PCR program is 3 min of denaturation at 94°C, 25 cycles of 30 s at 94°C, 30 s at 56°C, 1 min at 72°C, and followed by an 10 min extension at 72°C. After amplification, the PCR products were loaded for electrophoresis in 1% agarose gels.

### Enzyme activity assay

To identify the Res synthetic activity of the protein coded by *PNRS1*, a 4CL gene of *At4CL2* (GenBank: NM113019) was cloned from *Arabidopsis* using primers of F: 5′-CGGATCCATGACGACACAAGATGTGATAG-3′ and R: CCCTCGAGGTTCATTAATCCATTTGCTAGT-3′. After two prokaryotic expression vectors of pET-28a-PNRS1 and pMAL-c2x-At4CL2 were constructed, they were co-transformed into *E*. *coli* BL21(DE3), and the positive transformants were screened and identified by PCR and SDS-PAGE after culturing with 1 mM IPTG for 12 h. The positively transformed strain which expressed both recombinant proteins was used to synthesize Res in LB medium containing 2 mM *p*-coumaric acid and 0.1 mM IPTG for 48 h. The culture was collected and centrifuged at 12,000 rpm for 15 min, and 50 μl of 1 N HCl was added to 1 ml supernatant before it was kept at -20°C overnight. Equal volumes of acetic ether were used to extract Res from the thawed solution, and the precipitate was dissolved with 100 μl methanol after volatilization of the solution [33]. The extraction was kept at -20°C until HPLC analysis.

### Resveratrol extraction

The Res extraction processes were carried out following the method of Tang et al. [9] with moderate modification. Three grams of two-week-old T_3_ *PNRS1* transgenic or wild-type rice seedlings were collected. After the materials were ground to a fine powder and transferred into a flask with 20 ml 95% ethanol, the liquids were treated by microwave (500W) for 4 min, and then were shaken overnight at room temperature. After being centrifuged for 15 min at 5000 rpm, the supernatants were collected, and another 20 ml 95% ethanol was added to the precipitates for further extraction. Finally, the two supernatants were combined for volatilization. The remaining materials were then fully dissolved in 3 ml of methanol, and the extracted solutions were stored at -20°C for HPLC analysis. The extracts of seeds and white grains of transgenic or wild-type rice were extracted as described above, using one gram of each as starting materials.

### HPLC analysis

The extracts were filtered through a 0.45 μm membrane filter and analyzed in a Waters 2996-PDA HPLC system using a Waters Atlantic C_18_ column (4 μm, 150 mm×3.9 mm) at room temperature. H_2_O-acetonitrile was used as the mobile phase (acetonitrile:H_2_O = 25:75, flow rate 0.7 ml/min), and 306 nm detection wavelength was used. The commercial *trans*-resveratrol (3,4′,5-trihydroxy-*trans*-stilbene 99% GC; Sigma-Aldrich, St. Louis, MO) was dissolved in methanol and used as the standard sample. The peaks of extracted *trans*-resveratrol were identified during HPLC by their retention times compared with the standard sample, and the amounts were quantified by their peak areas.

### UV-C and dark treatment

Two-week-old rice seedlings and six-week-old rice leaves were used in this experiment. Seedlings were incubated in basal medium, and leaf fragments (2.5 cm^2^) were detached and incubated with water in petri dishes covered by preservative film. For UV-C treatment, an 8 W UV-C lamp (254 nm) was used, with the rice seedlings placed 15 cm below the UV-C lamp. After being irradiated for 1 h, the seedlings were put back into the growth chamber to continue growth for 24 h at 28°C in the dark [9]. The method of solely dark treatment was that of Jan et al. [34] with slight modification. The materials were all cultured in a growth chamber without light at 28°C for 6 days. The treated rice seedlings or leaf pitches were separately harvested and used for the following experiments.

### DAB coloration

The DAB dyeing method was used as Thordal-Christensen et al. [35], and 3,3′-diamino benzidine tetrahydrochlooride (DAB 4HCl) was purchased from Amresco. The DAB was dissolved in 0.2 M phosphate buffer (PH 7.0) to a final concentration of 1 mg/ml. Leaf fragments were dropped into the staining solution in the dark at 28°C for 8 h. Then, 95% ethanol was added and used for removing the chlorophyll in a boiling water bath.

### Determination of chlorophyll contents

The leaf fragments (0.2 g each) were collected and ground to a fine powder, and then 20 ml 95% ethanol was added to extract chlorophyll at 4°C. After 1 h extraction, the liquid samples were centrifuged for 15 min at 5000 rpm, and the supernatant was used for spectrophotometer determination. The absorption values of the extracts were measured at 665 nm, 649 nm, and 470 nm, and chlorophyll contents were calculated according to the Lichtenthaler formula [36].

### Data analyses

All measurements were performed in triplicate and the data were presented as mean ± standard deviation (SD). The statistical analysis was conducted by one-way analysis of variance (ANOVA) for evaluating the variances in the whole groups under a single condition, and Student t-test for comparing the average of a specific group to the total average.

## Results

### Enzymatic determination of RS coded by *PNRS1*

**Fig 1:**
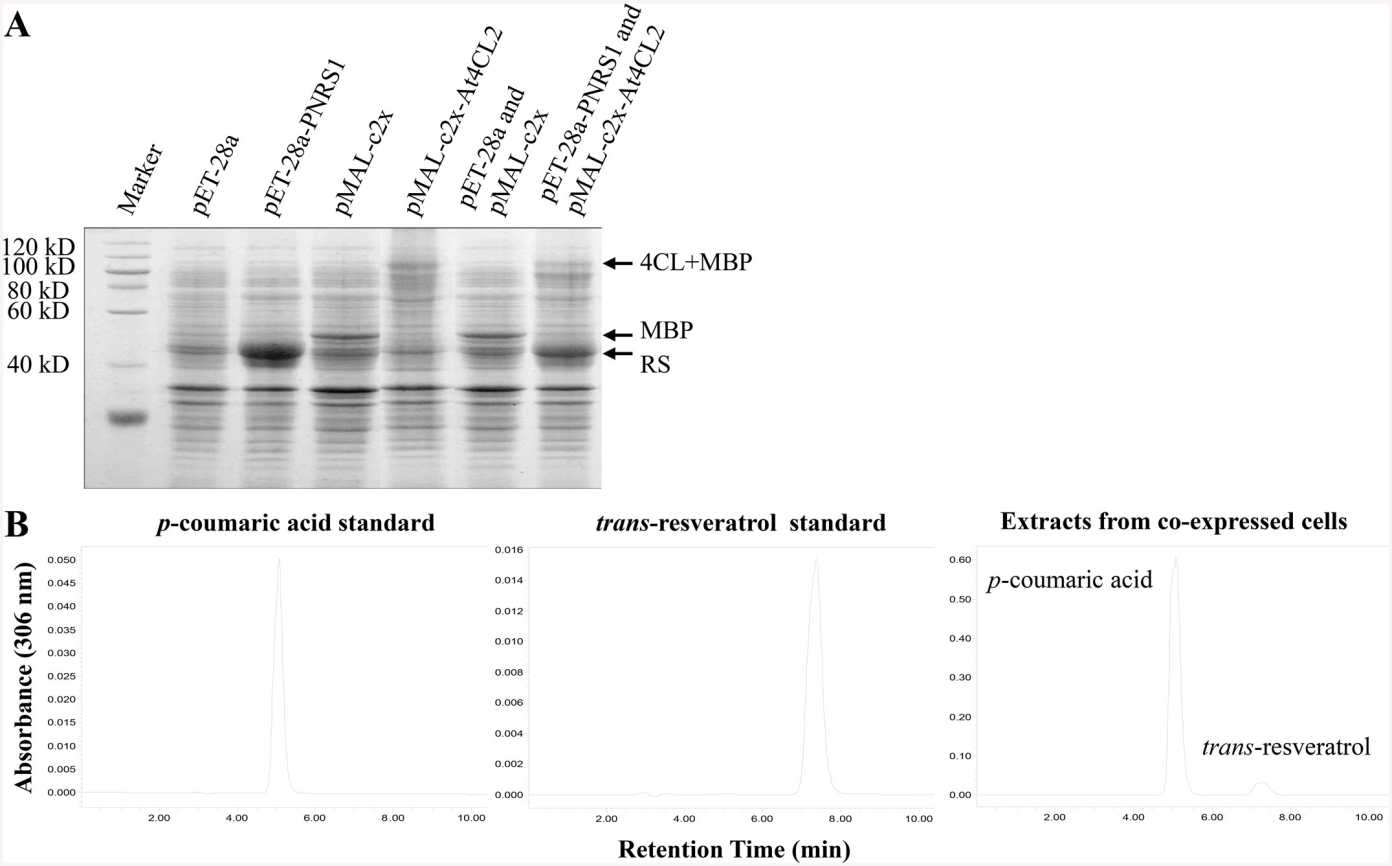
Determination of RS enzyme activity. **A** Co-expression of *PNRS1* and *At4CL2* in *E*. *Coli*. Two vectors, pET-28a and pMAL-c2x, were used for prokaryotic expression, and the cell lysates of BL21(DE3) transformed with empty or gene recombinanted vectors were analyzed by SDS-PAGE. Molecular weight standard of protein was shown on the left. The proteins of RS coded by *PNRS1* (46 kD), MBP (50 kD) and MBP fused 4CL code by *At4CL2* (103 kD) were indicated on the right. **B** HPLC identification of Res production in *PNRS1* and *At4CL2* co-expressed *E*. *coli*. Both the peaks of *p*-coumaric acid and *trans*-resveratrol were obtained in the chromatogram of extracts from the co-cultured medium, which were detected at 306 nm under the same chromatographic conditions.

To detect the enzymatic activity of RS, the two substrates of coumaryl Co-A and malonyl Co-A are required. While coumaryl Co-A is absent in *E*. *coli*, *At4CL2* was cloned for converting *p*-coumaric acid to coumaryl Co-A. The results of SDS-PAGE were shown in Fig 1A, 6× His tag fused RS (46 kD) or Maltose binding protein (MBP) fused 4CL (103 kD) emerged in the cell lysate of *E*. *coli* transformed with pET-28a-PNRS1 or pMAL-c2x-At4CL2, compared with the protein brands of related empty vectors. Both the proteins were detected in the cell lysate of pET-28a-PNRS1 and pMAL-c2x-At4CL2 co-transformed *E*. *coli*, while only MBP was induced in the cell lysate of pET-28a and pMAL-c2x co-transformed *E*. *coli*. Then the *PNRS1* and *At4CL2* co-expressed strain was used for Res synthesis analysis. HPLC results showed that both *p*-coumaric acid and *trans*-resveratrol could be detected in extracts from the medium of the co-expressed strain cultured for 48 h, determined by the same retention time of each standard (Fig 1B). Thus, the protein coded by *PNRS1* from peanut revealed its catalytic activity of RS.

### Molecular analysis of *PNRS1* in transgenic rice

**Fig 2:**
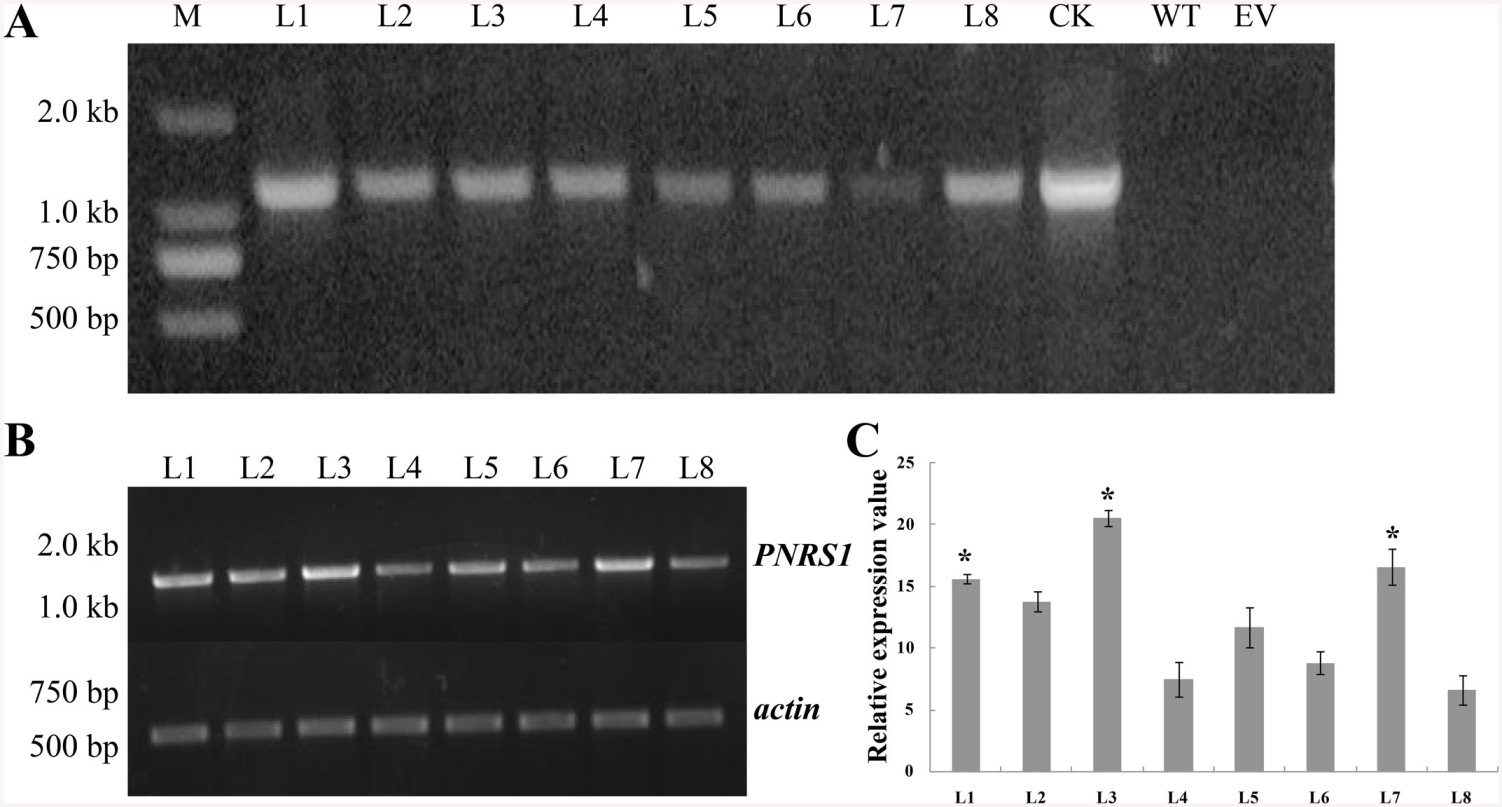
Molecular identification of *PNRS1* transgenic rice. The weight of DNA marker was shown on the left. **A** PCR identification of the integration of *PNRS1* in the rice genome. The genomic DNA from *PNRS1* transgenic rice lines of L1–L8 (L1–L8), from empty-vector transformed rice (EV), from wild-type rice “Shengdao 13” (WT), and the plasmid pCA1300-Ubi-PNRS1 (CK), were used as the templates for PCR amplification. **B** Semi-quantitative RT-PCR of *PNRS1* in transgenic rice lines L1–L8, using the internal control of *actin*. **C** The relative expression levels of *PNRS1* in transgenic lines L1–L8. Bars represent the mean value ± SD from three independent experiments (10 seedlings each).* the value significantly higher than the average value analyzed by student t-test (p<0.01).

In the T_3_ generation, the presence of *PNRS1* in transgenic rice was primarily screened by basta, and then confirmed by genomic PCR (Fig 2A). Results showed that the estimated band (1.2 kb) of *PNRS1* appeared after PCR amplification, which indicated that the peanut RS gene *PNRS1* had been integrated into eight independent transgenic rice lines. Semi-quantitative RT-PCR analysis was performed to determine the transcription of *PNRS1* in transgenic rice L1–L8. Total RNA was isolated from two-week-old rice seedlings grown under normal conditions. As shown in Fig 2B and 2C, The *PNRS1* transcription levels of L1, L3, and L7 were higher than those of other lines. The above mentioned rice transgenic lines of L1–L8, which were proved to be successively and stably transformed with *PNRS1*, were then selected for further analysis.

### HPLC analysis of trans-resveratrol in *PNRS1* transgenic rice seedlings and seeds

**Fig 3:**
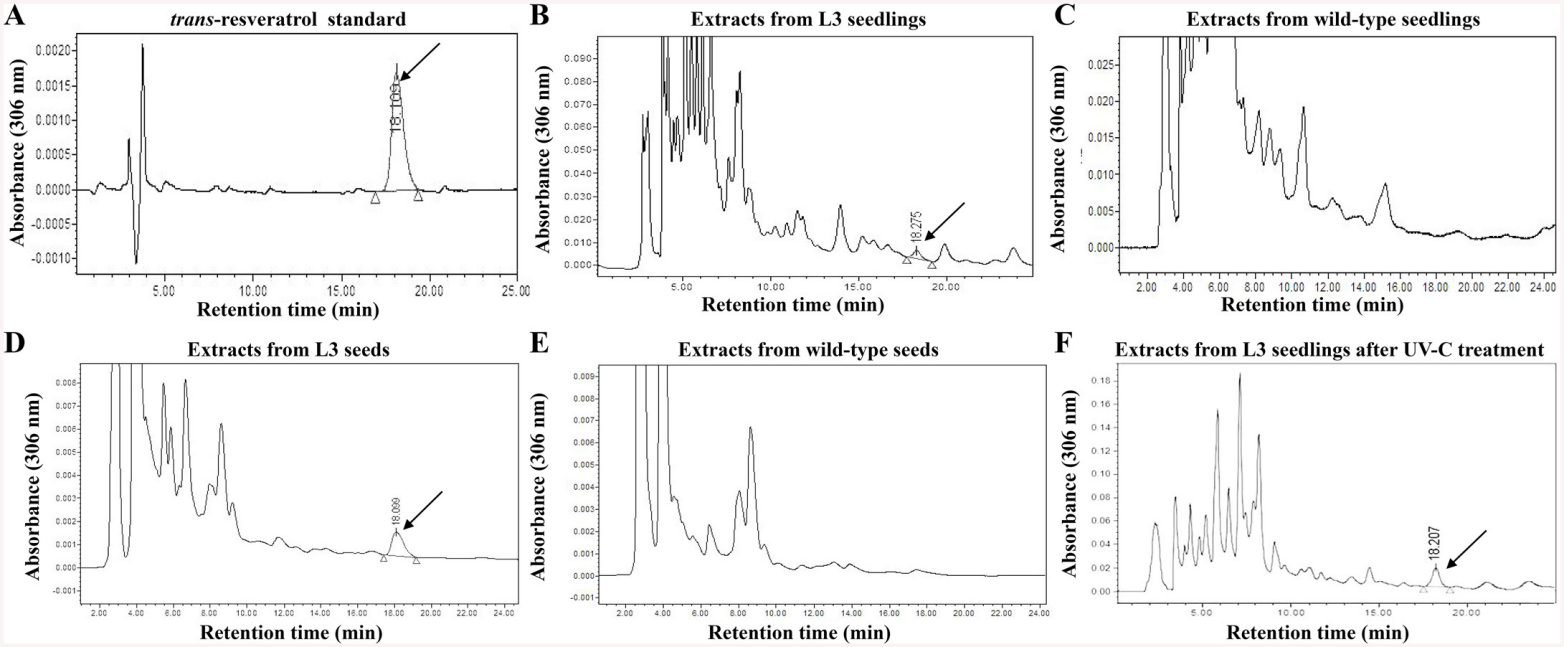
Identification of trans-resveratrol in the seedlings and seeds of transgenic and wild-type rice by HPLC analysis. The chromatograms of standard *trans*-resveratrol (**A**), the extracts from L3 seedlings (**B**), wild-type rice seedlings (**C**), L3 seeds (**D**), wild-type rice seeds (**E**) and L3 seedlings treated by UV-C (**F**, introduced in the later section), were detected at 306 nm under the same conditions. The marker “↙”indicates the peak of *trans*-resveratrol.

The synthesis and accumulation of *trans*-resveratrol was the focus of our research instead of other kinds of newly synthesized metabolites mentioned above. The detection of HPLC showed that one new peak emerged in the chromatogram in the extracts of L3 seedlings (Fig 3B), which did not exist in those of wild-type seedlings (Fig 3C). The retention time of the newly emerged peak was about 18 min, which was in accordance with that of *trans*-resveratrol standard (Fig 3A), indicating that the newly emerged peak represents the production of *trans*-resveratrol in L3. When HPLC analysis was performed on the extracts from rice seeds of L3 and WT, the chromatograms also showed similar results with those of seedlings, in which the Res specific peak appeared only in the extracts of transgenic rice (Fig 3D and 3E). And the peaks represent Res in the chromatograms of seeds were purer with less noise or background than those of the seedlings (Fig 3B and 3D). Furthermore, as polished grains without seed coats are normally used in people’s daily diet, the contents of Res in white grains were also been detected, and the results are almost identical to that of seeds.

### Quantification of trans-resveratrol in transgenic rice seedlings and seeds

The contents of *trans*-resveratrol in transgenic rice lines were quantified by the ratio of the specific peak areas and the concentration of the standard sample used [9]. As shown in Table 1, there were about 0.280 ± 0.021 μg/g fresh weight (FW) to 0.697 ± 0.028 μg/g FW of *trans*-resveratrol detected in transgenic rice seedlings, while the concentration of *trans*-resveratrol in transgenic rice seeds varied from 1.056 ± 0.042 μg/g dry weight (DW) to 3.028 ± 0.024 μg/g DW. The concentration of Res in polished white grains was also quantified, and the results showed that there were about 0.621 ± 0.024 μg/g DW to 1.084 ± 0.014 μg/g DW of *trans*-resveratrol accumulated. These results indicated that the new metabolite of *trans*-resveratrol could be accumulated in different parts of *PNRS1* transformed rice.

**Table 1:** Contents of trans-resveratrol in different parts of transgenic rice detected by HPLC.

Line	Seedlings (μg/g FW)	Seeds (μg/g DW)	Polished white grains (μg/g DW)
**WT**	0.082 ± 0.01^a^	0.000±0.0^a^	0.000±0.0^a^
**EV**	0.079 ± 0.012	0.000±0.0	0.000±0.0
**L1**	0.557 ± 0.013	1.667 ± 0.032	0.923 ± 0.031
**L2**	0.326 ± 0.005	1.332 ± 0.032	0.769 ± 0.018
**L3**	0.697 ± 0.028^b^	3.028 ± 0.024^b^	1.084 ± 0.014^b^
**L4**	0.280 ± 0.021	1.221 ± 0.07	0.621 ± 0.024
**L5**	0.372 ± 0.029	1.496 ± 0.015	0.875 ± 0.033
**L6**	0.343 ± 0.005	1.056 ± 0.042	0.785 ± 0.018
**L7**	0.542 ± 0.022	1.557 ± 0.103	0.932 ± 0.024
**L8**	0.352 ± 0.016	1.187 ± 0.08	0.739 ± 0.013

Each value represents the mean ± standard deviation (SD) from three independent experiments.

^**a**^ Significant variance existed among the groups of each kind when analyzed by one-way ANOVA (p<0.01).

^**b**^ The data showed the highest value from total averages in each kind of sample when analyzed by Student’s t-test (p<0.01). WT: wild-type rice; EV: empty vector transformed rice; L1–L8: different transgenic lines of rice.

### UV-C radiation on transgenic and wild-type rice

**Fig 4:**
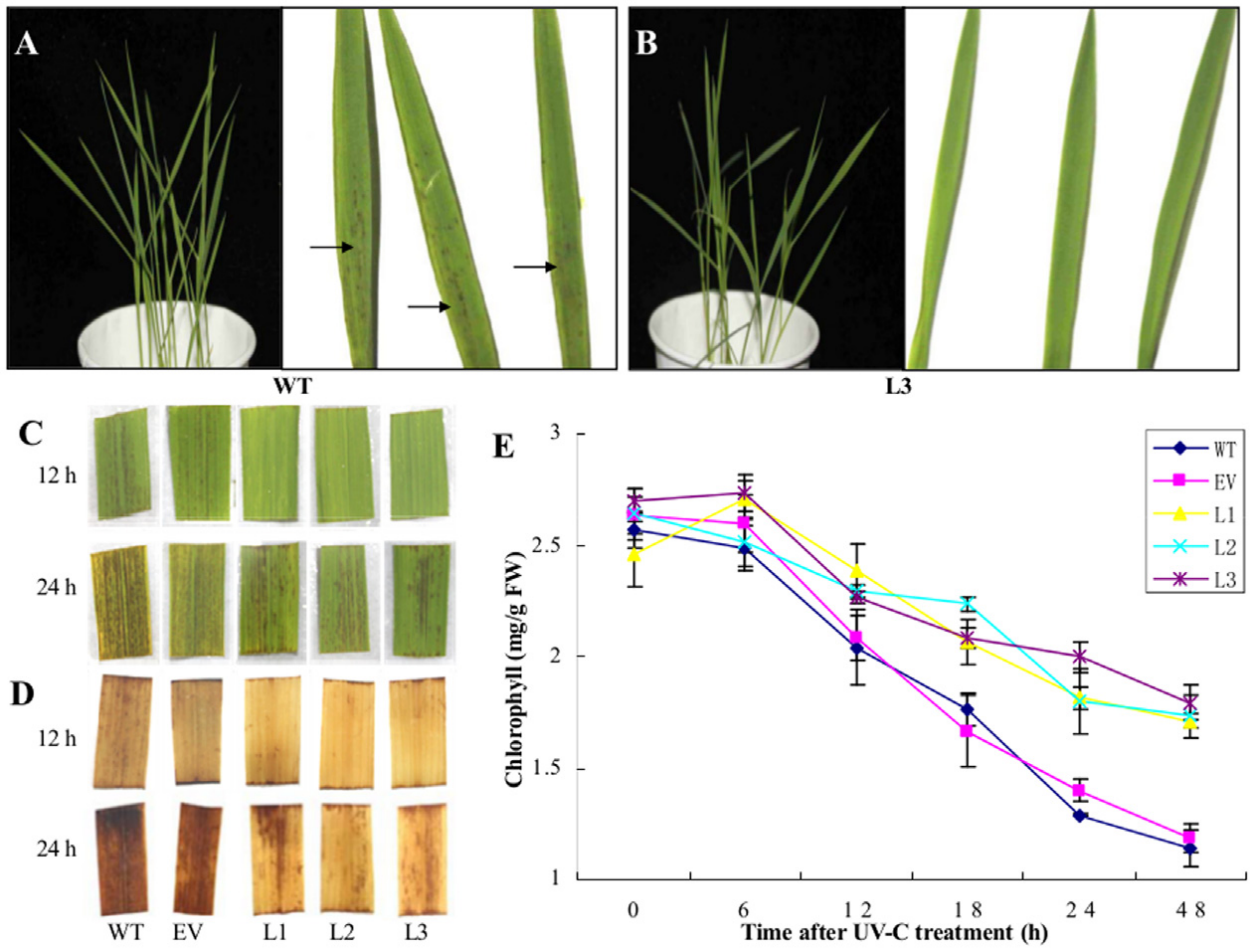
Different phenotypes of wild-type and RS transgenic rice under UV-C treatment. **A**, **B** two-week-old wild-type rice and L3 seedlings were treated by short UV-C radiation, separately. “→” indicates the brown patches appeared on the rice leaves. **C** six-week-old rice leaves were treated by UV-C radiation. **D** DAB coloration using fragments of six-week-old rice leaves under different UV-C treatment conditions. 12 h and 24 h were the time after UV-C treatment. **E** Chlorophyll determination of six-week-old rice leaves after treated by UV-C radiation. Each value represents the mean ± SD from three independent experiments (0.2 g leaves from 10 plants each). WT wild-type rice “Shengdao 13”; EV empty-vector transformed rice; L1–L3 *PNRS1* transgenic rice lines.

UV radiation has been reported as an environmental stress factor that can induce leaf chlorosis and necrosis by causing oxidative stress in plants [9, 30]. The phenotype of two-week-old wild-type and RS transgenic rice seedlings in response to UV damage were observed. After the materials underwent 1 h of UV-C radiation and then were kept in the dark for another 12 h recovery, brown patches of the UV-C injury appeared around the central vein on the leaves of the wild-type rice (Fig 4A), but it was almost invisible on the leaves of L3 (Fig 4B). When the leaf fragments from six-week-old wild-type and RS transgenic rice were used for the same treatment, similar phenomena were observed after 12 h and 24 h treatments. The rusty spots emerged on wild-type leaves earlier and more seriously than those on RS transgenic leaves (Fig 4C). DAB coloration also showed that the DAB staining was much deeper in the leaf fragments of the wild-type than those of RS transgenics (Fig 4D), which indicated that the accumulation of H_2_O_2_ were severer in wild-type leaves than those in RS transgenic leaves. The changes of chlorophyll contents of rice leaves were also evaluated after UV-C radiation, which displayed differences at 12 h, and this gap continued to increase significantly at 18 h and 24 h, after UV-C treatment (Fig 4E). These results suggested that UV-C caused lesion was slighter in *PNRS1* transgenics than those in wild-type rice, and this might be related to the production of Res.

### Dark treatment on transgenic and wild-type rice

**Fig 5:**
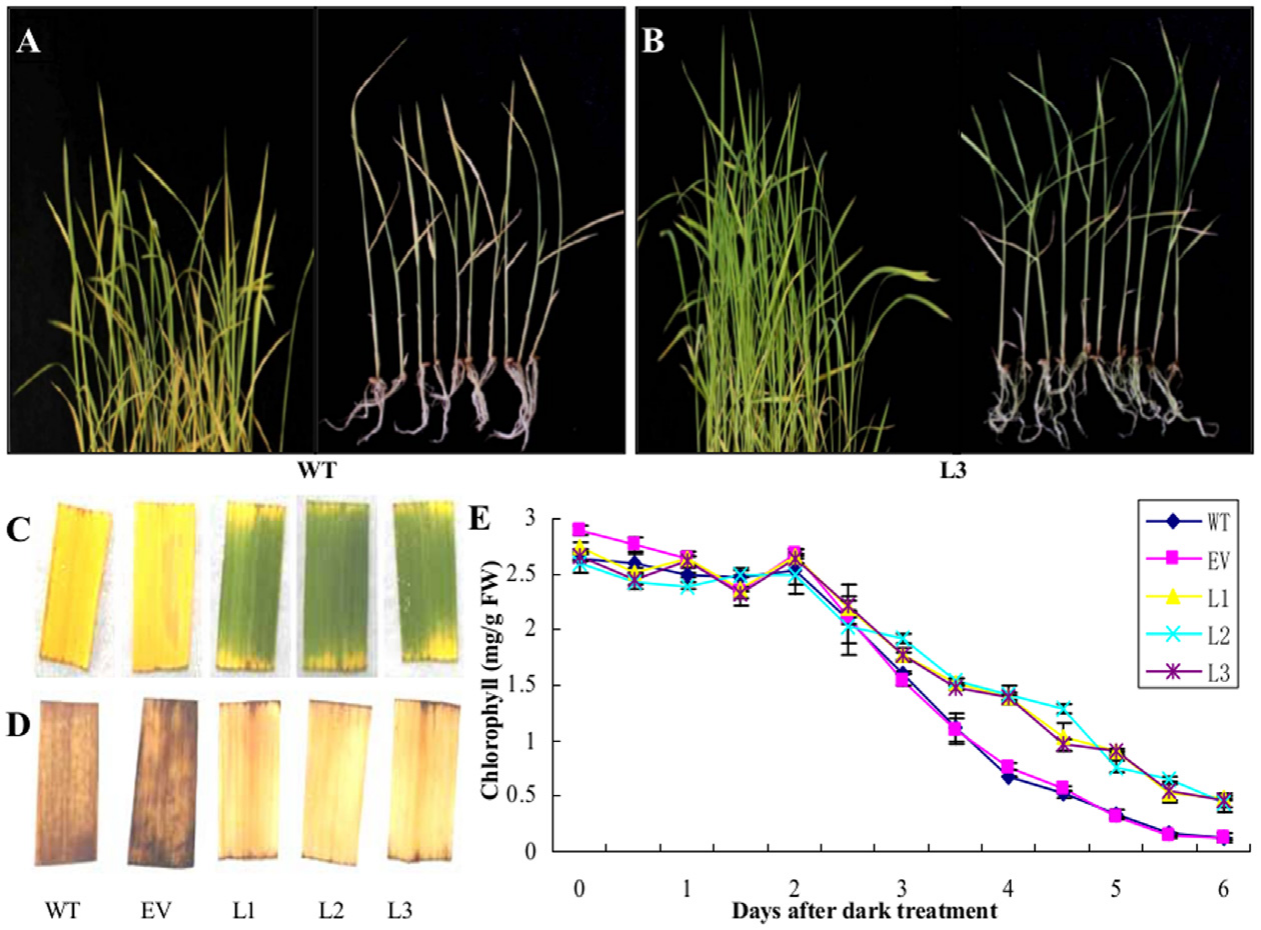
Different phenotypes of wild-type and RS transgenic rice under dark treatment. **A**, **B** two-week-old wild-type rice and L3 seedlings were treated solely in the dark for 6 days. **C** six-week-old rice leaves incubated solely in the dark for 6 days. **D** The 6 days dark treated six-week-old rice leaves were stained with DAB. **E** Changes of chlorophyll in six-week-old rice leaves after being treated solely in the dark. Each value represents the mean ± SD from three independent experiments (0.2 g leaves from 10 plants each). WT wild-type rice “Shengdao 13”; EV empty-vector transformed rice; L1–L3 *PNRS1* transgenic rice lines.

Absence of light has been reported as an elicitor for plant senescence, as well as the induction of etiolation and oxidative stress [34, 37]. When two-week-old seedlings of RS transgenic and wild-type rice were grown in the dark for 6 days, the seedlings of wild-type rice (Fig 5A) were withering and yellowing faster than those of L3 (Fig 5B). When treated under the same condition, the leaf fragments of six-week-old wild-type rice also displayed a more serious etiolation phenotype than those of transgenics (Fig 5C). When DAB coloration was performed on the corresponding leaf fragments, the results showed that DAB staining of wild-type leaves was deeper than those of RS transgenic leaves (Fig 5D), which indicated a severer accumulation of H_2_O_2_. The variation trends of total chlorophyll in leaves after dark treatment were in accordance with those of the corresponding phenotypes, and the dropping levels of chlorophyll were significantly slower in transgenics than those of wild-type rice and empty vector transformed rice(Fig 5E). These results revealed a delayed leaf senescence phenotype in *PNRS1* transgenic rice, which may be related to the antioxidant activity of the newly synthesized Res.

### Effects of UV-C and dark on the accumulation of trans-resveratrol

**Fig 6:**
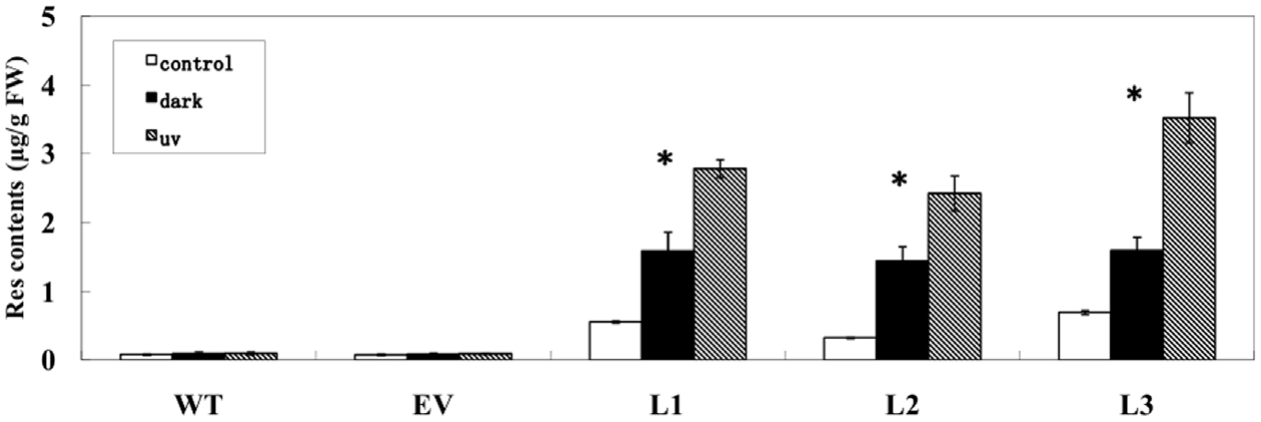
Detection of trans-resveratrol contents in transgenic rice lines grown under UV-C or dark treatments. The Data of Res content of each line detected under normal grpwth condition, as shown in Table 1, was used as relevant control. Bars represent the mean values ± SD from three independent experiments (30 seedlings each). Statistical analysis was performed by using one-way ANOVA. * Significant variances existed under different conditions in each transgenic line (p<0.01). WT wild-type rice “Shengdao 13”; EV empty-vector transformed rice; L1–L3 *PNRS1* transgenic rice lines.

The accumulation of Res was reported to be increased under UV radiation in grape, peanut, and RS gene transgenic tobacco [8, 9, 17]. Furthermore, other ROS related elicitors, such as fungal infection, ozone exposure, and wounding were also reported to accelerate the synthetic processes of Res in plants [38]. Three transgenic rice lines of L1, L2 and L3, together with empty-vector transformed line and wild-type rice, were used to detect the accumulation of Res under UV-C or dark treatment. As shown in Fig 6, consistent with the chromatograms (Fig 3B and 3F), the contents of *trans*-resveratrol in three transgenic lines were all significantly elevated when treated by UV-C or dark. Compared with the relevant control under UV-C treatment, Res contents increased 5–8 times to 2.784 ± 0.128 μg/g FW, 2.425 ± 0.253 μg/g FW, and 3.524 ± 0.366 μg/g FW in L1, L2 and L3 transgenic rice seedlings seperately. When treated by dark, there were about 1.59 ± 0.27 μg/g FW, 1.446 ± 0.205 μg/g FW, and 1.597 ± 0.194 μg/g FW of *trans*-resveratrol in L1, L2 and L3 transgenic rice seedlings, which increased 3–4 times compared with that of the relevant control.

### Physiogical and nutritional indexes in wild-type and transgenic rice

To identify the influence of recombinanted gene of *PNRS1* to physiogical and nutritional phenotypes of transgenic rice, the indexes of plant height, flag leaf length, effective tiller number, spike length, spike weight, thousand-grain weight, and the content of amylose and total protein in white grains were measured and detected. And the results obtained indicated that there have no much significant differences between wild-type rice and those of transgenics (S1 and S2 Figs).

## Discussion

The polyphenolic structure of Res indicates its basic antioxidant activity, and the ability to prevent cells from ROS related damages [4]. Previous studies had shown that the accumulation of Res in host plants was correlated with some biotic or abiotic stresses [1], suggesting that Res may be involved in the plant defense responses. On the other hand, the extracts of Res from plants, such as grape, peanut, and giant knot weed, together with the Res that exists in red wines or people’s daily diet, had been revealed to be beneficial for health and fitness [39]. Thus, it would be an interesting and valuable project to improve and optimize the content and purity of Res in crops and plant materials by molecular biological and bioengineering techniques.

### The production of Res is various in transgenic plants

In previous research, investigators introduced RS into various fruits and vegetables, where Res or its derivates could be detected, such as in kiwifruits [20], apple [40], oilseed rape [41], pea [42], lettuce [11], tomato [19], and potato [23]. A few investigators had also tried to transform the RS gene into crops [27]. The final results of the above investigation showed that, there were different type and amount of newly metabolites synthesized, although operated with the similar heterogenous transformation method.

HPLC, which is a simple, fast, accurate and efficient biochemical detection method, had been used to detect natural or artificial Res and its derivatives in plants [9, 19]. Due to the existence of endogenous modification enzymes in host plants, especially glycosylase, the metabolic products resulting from the transformed RS gene were mostly detected as some derivatives of Res [14], e.g. piceid (resveratrol-3-O-β-D-glucopyranoside), or even not confirmed [16, 43]. And as piceid, the soluble form of Res, had once been thought to be not as active as Res [44], the production of *trans*-resveratrol was the only metabolite concerned with in this research (Fig 1), and its contents were detected in transgenic rice seedlings, seeds and polished white grains by HPLC analysis with *trans*-resveratrol standard (Fig 3).

It had been reported that the concentration of Res in transgenic plants varied according to the different receptor plants, driving promoters, or RS gene origins [14]. 2.586 μg/g FW, 2.7 μg/g FW, 0.79–15.8 μg/g FW and 56.4 μg/g FW of *trans*-resveratrol was reported in transgenic grape [45], hops [46], tomato [19] and lettuce [11] respectively, in which the RS gene was driven to express by the CaMV 35S promoter. As shown in Table 1, the highest level of trans-resveratrol in L3 was 0.697 μg/g FW in seedlings and 3.028 μg/g DW in seeds, which were grown under normal condition and driven by the Ubi promoter. Considering the concentration of 1.9 μg/g DW Res in transgenic rice seeds reported by Baek et al. [27], the contents of Res in our materials are comparable.

### The anti-oxidant activity of Res was supplied to plants

In plants, researchers were concerned with investigations of Res to enhance plant resistance to biotic and abiotic stresses [1, 3]. UV radiation and dark induced to leaf senescence had been extensively studied in numerous plants, and the increase of the ROS level was thought to be the direct elicitor in causing cell lesions [30, 34, 37]. Although a complex regulation mechanism was involved, the use of some antioxidant compounds, such as polyphenols would reduce the damage [47]. And reports also suggested that the accumulation of Res in tomato could lead to the increase of enzyme activities such as ascorbic acid, glutathione, and antioxidants, as well as to inhibit other enzyme activity such as cyclo-oxygenase-2 [6]. Exogenous applied Res to peanut plants before UV-C treatment could mitigate the damage symptoms of rusty spots and leaf wilt [9]. The external application of Res to apples could also delay their decay process during fruit storage [29]. Res could also cope with the ROS stress in transgenic plants by reducing the damage of cell membranes and maintaining the stability of the cells [4, 38]. Therefore, the endogenous accumulation of Res, which was produced by the heterogeneous RS gene, was also presumed to have some protective roles against abiotic stress in transgenic rice.

The results derived from our experiments showed that the contents of Res were significantly increased 5–8 fold under UV-C and 3–4 fold under dark in *PNRS1* transgenic rice seedlings, compared to those grown under normal conditions (Table 1). The shape of brown patches and chlorosis were the obvious corresponding signs that emerged on the rice leaves after UV radiation and dark induced leaf senescence [34, 48]. Our results revealed that the transgenic rice did not show as critical damage symptoms as the wild-type rice when subjected to the two stresses, (Figs 4 and 5). Furthermore, the accumulation of Res had no significant influence on other physiological indexes of rice, such as plant height, leaf length and thousand-grain weight (S1 Fig). These observations indicated that the peanut RS gene *PNRS1* could be used as a candidate gene for heterogenic transformation in promoting Res synthesis and protecting acceptor plants from abiotic stresses. Considering the previous research progresses together with the results mentioned above, it can almost be assured that Res, based on its chemical structure, is involved in the process of ROS removal, which will play a role in improving resistance against biotic and abiotic stresses.

### Res enriched rice could be valuable

Because of the attention on improving the nutritional value of crops and the health benefit properties of Res [38], it is necessary and urgent to broaden the resources of Res into daily diets so that people and animals can widely and directly get a healthy supply. But up to now, very few successful attempts related to this aspect have been made. Red wines were known to be an important source containing Res richly, and researchers had further transformed RS gene into hops for the brewing industry in order to improve the quality of beer [46]. RS gene transgenic tomatoes showed higher antioxidant activity and better fitness effect when fed to inflammation rats, compared with those fed by the wild-type tomatoes [2, 6]. Experiments on Res-enriched rice grains showed some benefits in improving anti-metabolic syndrome activity and inducing *Sirt1*, a factor relating to cell proliferation, aging, apoptosis, and metabolism [27, 49]. And our results showed that the enrichment of Res didn't affect the levels of basal nutrients (amylose and total protein) in rice grains (S2 Fig). As well, the Ubi promoter was a safe and efficient promoter in plant genetic engineering [31], and the selectable marker of bar gene could be removed for further commercial and agricultural use [50]. Certainly, more evidences of the relationship between Res-enriched crops and health care, and investigations for the application of Res accumulated transgenic food in our daily life, still need to be deeply and intensively studied.

## Supporting Information

S1 Fig Determination of physiological indexes in wild-type and transgenic rice. Thirty plants of wild-type rice or each transgenic line were used for determination of plant height (**A**), flag leaf length (**B**), effective tiller number (**C**), spike length (**D**), spike weight (**E**) and thousand-grain weight (**F**). Bars represent the mean values ± SD. Statistical analysis was performed by using one-way ANOVA. WT wild-type rice “Shengdao 13”; EV empty-vector transformed rice; L1–L3 *PNRS1* transgenic rice lines.
(TIF)

S2 Fig Detection of amylose and total protein levels in wild-type and transgenic rice grains. The continuous spectrum and fixed grating analyzer DA7200 (Perten, Sweden) was used to detect the relative level of amylose (**A**) and total protein (**B**) in polished grains (100 g each), under a same moisture content, triplicately. Bars represent the mean values ± SD. Statistical analysis was performed by using one-way ANOVA. WT wild-type rice “Shengdao 13”; EV empty-vector transformed rice; L1–L3 *PNRS1* transgenic rice lines.
(TIF)
