# Supplementary figures and images for: Evaluating the Effect of Expressing a Peanut Resveratrol Synthase Gene in Rice
Source: PLoS One. 2015 Aug 24;10(8):e0136013. doi: 10.1371/journal.pone.0136013 (PMC4547805; doi:10.1371/journal.pone.0136013)

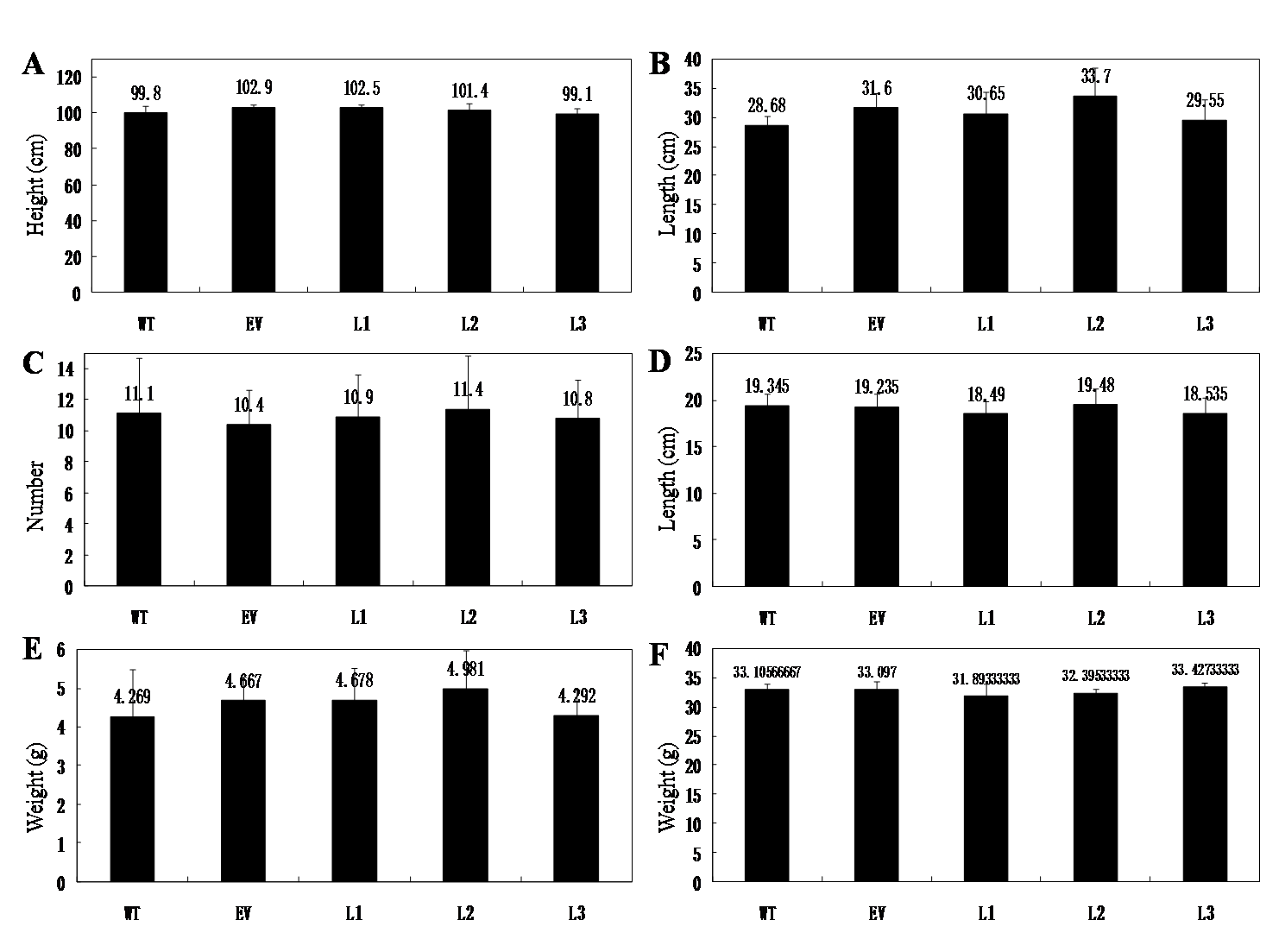

Supplement: S1 Fig — Thirty plants of wild-type rice or each transgenic line were used for determination of plant height (A), flag leaf length (B), effective tiller number (C), spike length (D), spike weight (E) and thousand-grain weight (F). Bars represent the mean values ± SD. Statistical analysis was performed by using one-way ANOVA. WT wild-type rice “Shengdao 13”; EV empty-vector transformed rice; L1–L3 PNRS1 transgenic rice lines. (TIF) [file pone.0136013.s001.tif]

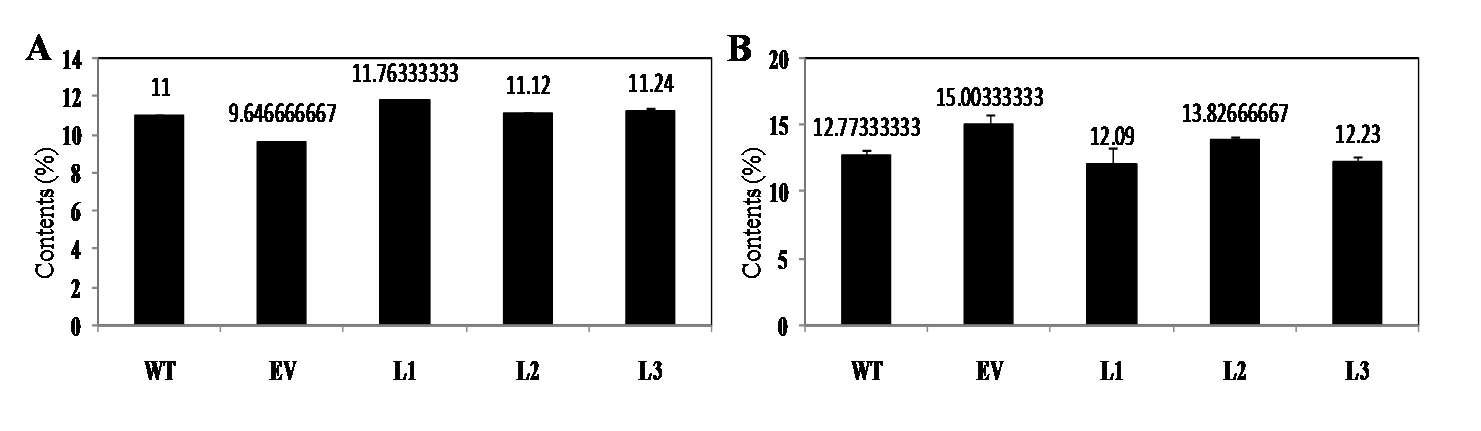

Supplement: S2 Fig — The continuous spectrum and fixed grating analyzer DA7200 (Perten, Sweden) was used to detect the relative level of amylose (A) and total protein (B) in polished grains (100 g each), under a same moisture content, triplicately. Bars represent the mean values ± SD. Statistical analysis was performed by using one-way ANOVA. WT wild-type rice “Shengdao 13”; EV empty-vector transformed rice; L1–L3 PNRS1 transgenic rice lines. (TIF) [file pone.0136013.s002.tif]
